# Person-Centered, Physical Activity for Patients with Low Back Pain: Piloting Service Delivery

**DOI:** 10.3390/healthcare4020028

**Published:** 2016-05-18

**Authors:** Saul Bloxham, Phil Barter, Slafka Scragg, Charles Peers, Ben Jane, Joe Layden

**Affiliations:** 1 Department of Health Sciences, University of St Mark and St John, Plymouth PL11 8BH, UK; bjane@marjon.ac.uk (B.J.); jlayden@marjon.ac.uk (J.L.); 2 London Sport Institute, Middlesex University, London NW4 4BT, UK; p.barter@mdx.ac.uk; 3 Plymouth Hospitals NHS Trust, Plymouth PL6 8DH, UK; slafka.scragg@nhs.net; 4 Plymouth Community Back Pain Service, Stoke Surgery, Belmont Villas, Stoke, Plymouth PL3 4DP, UK; charles.peers@nhs.net

**Keywords:** low back pain, physical activity, disability, self-management, well-being, physical fitness

## Abstract

Low back pain (LBP) is one of the most common and costly conditions in industrialized countries. Exercise therapy has been used to treat LBP, although typically using only one mode of exercise. This paper describes the method and initial findings of a person-centered, group physical activity programme which featured as part of a multidisciplinary approach to treating LBP. Six participants (aged 50.7 ± 17 years) completed a six-week physical activity programme lasting two hours per week. A multicomponent approach to physical activity was adopted which included aerobic fitness, core activation, muscular strength and endurance, Nordic Walking, flexibility and exercise gaming. In addition, participants were required to use diary sheets to record physical activity completed at home. Results revealed significant (*p* < 0.05) improvements in back strength (23%), aerobic fitness (23%), negative wellbeing (32%) and disability (16%). Person’s Correlation Coefficient analysis revealed significant (*p* < 0.05) relationships between improvement in perceived pain and aerobic fitness (*r* = 0.93). It was concluded that a person-centered, multicomponent approach to physical activity may be optimal for supporting patients who self-manage LBP.

## 1. Introduction

Low back pain (LBP) is a major health concern in Western countries and is associated with high medical expenditure, work absence [1,2,3] and is the most common musculoskeletal condition [4,5,6]. Sixty to eighty percent of adults are likely to experience LBP [7,8] with 16% of adults in the United Kingdom (UK) consulting their general practitioner every year [9]. Back pain costs the UK National Health Service £1.3 million every day [1] and results in 12.5% of all work absence in the UK [10]. Low back pain is multifactorial and can have a significant effect on patients’ quality of life. Completing routine domestic tasks such as vacuum cleaning, lifting, bending, sitting, twisting, pulling and pushing, repetitive work, static postures and opening doors can become severely restricted [11,12]. Contributory factors to LBP have included heavy physical work, physical fitness, social class, occupation and employment status, drug and alcohol use and smoking history [13,14] yet diagnosing the specific pathological or neurological cause of LBP in individual cases is often not possible [15].

In recent years, exercise therapy has been explored to treat LBP [16,17,18,19]. It can be delivered to a group of patients [20] and is more cost effective than individual treatment [21]. The term exercise therapy encompasses a range of different approaches (aerobic, strengthening and flexibility exercises) for which the evidence provides varying degrees of support. Studies suggest that flexibility is not correlated with measures of pain and disability [16], and those that focus upon spine flexibility have often yielded negative results [22]. In contrast, the use of strengthening and stabilising exercises has been shown to be more effective than General Practitioner treatment [23]. A growing body of research has endorsed the use of endurance training to reduce LBP [17,18,19], as significant reductions in pain intensity, disability and psychological strain have been highlighted.

Previous studies into LBP have focused on specific outcomes of muscular strength or endurance [18], yet few appear to have assessed the effectiveness of LBP exercise programmes which incorporate a range of approaches and outcome measures.

To date, the majority of research into exercise therapy as a treatment for LBP has centered on delivering monodisciplinary interventions that have focused on improving specific outcomes such as strength of the lumbar stabilizing muscles [23], functional range of motion of the lumbar spine [22] or aerobic fitness [18,19]. As approximately 85% of cases are non-specific [15] it is unlikely that one particular approach to exercise therapy can facilitate significant improvements in LBP.

At present, there is a paucity of research that explores the effectiveness of person-centered (bio-psycho-social), multicomponent exercise therapy interventions for the treatment of LBP. This paper describes methods and initial findings from a six week multicomponent physical activity programme aimed at improving physical fitness, physical activity, disability and psychological wellbeing of non-specific LBP patients.

## 2. Materials and Methods

According to best practice, the Local Health Authority had commissioned a multi-disciplinary team to treat sub-acute and chronic LBP consisting of Osteopathy, Cognitive Behavioral Therapy and exercise. The gym based exercise component had suffered from high drop-out, and as the local University, we were tasked with providing an alternative approach. The brief was to develop a low cost self-management style programme of exercise. We were instructed not to treat the cause of LBP, as specific causes had not been identified and patients had not responded to conventional treatment modalities. This pilot describes our approach taken to maximise adherence and promote self-management of LBP.

Four female and two male patients consented to partake in this pilot (mean age 51 years ± 17). All patients experienced non-specific LBP, and had been expressing symptoms for >3 months. Each patient was medically screened by their general practitioner and Physical Activity Readiness Questionnaire (PAR-Q) and informed consent were obtained. The stature (Leicester Height, Seca Limited, Birmingham, UK) and body mass (Weight Counting Scale, Seca Limited, Birmingham, UK) of the patients were 168.3 cm ± 8.8 cm and 87.7 kg ± 23.1 kg respectively. The instructors explained, demonstrated and supervised all physical activity undertaken by the group with support from student helpers studying for sport and health science related degrees. Each patient was fully informed of their right to withdraw from the programme at any time, or abstain from partaking in prescribed activities. The programme consisted of six weekly sessions lasting up to two hours. The sessions were divided into seven activity blocks to provide regular breaks and cater for patients’ needs as documented in Table 1.

### 2.1. Week One—Introduction

The session commenced with an introduction to the programme, followed by a team building activity and baseline testing. Anthropometric measures such as body mass, stature, body fat and lean muscle mass (Body Stat 1500 Body Composition Measuring Unit, Body Stat, Douglas UK) were obtained. Aerobic capacity was measured using the Chester Step Test (Assist Creative Resources, Wrexham, UK). A back strength dynamometer (Takei Physical Fitness Test, Niigata, Japan) was used to assess back and leg strength, and a hand grip dynamometer (Takei Physical Fitness Test, Niigata, Japan) was used to assess hand grip strength. The prone double straight leg raise test and the plank test were utilised to measure muscular endurance of the low back. Measures of flexibility of the low back, pelvis and hamstrings were recorded using a clinical goniometer (MIE Medical Research Limited, Leeds, UK) and a sit and reach box (Fitech, Southampton, UK). Patients completed a contract outlining the terms and conditions of the programme and Modified Oswestry Low Back Pain Disability (MODQ) and Well-being (WB12-Q) questionnaires. In addition, patients were encouraged to complete a “daily diary” sheet which included an adapted faces pain scale to indicate daily pain levels (1 = feels worst, 6 = feels best). These were subsequently used to inform weekly patient-instructor discussions completed at the start of every session alongside a recap and general introduction.

On the first week, an information booklet was provided to participants to help them complete a programme of home exercise and dietary advice to re-enforce educational themes covered during the sessions. The home exercises followed the weekly theme and the exercise prescription was individualised for each patient. This was informed by the weekly patent-instructor discussion, which encouraged a meaningful dialogue of trial, error and participant feedback. In addition, the context of how the exercises were completed was addressed to promote participant ownership and long-term adherence. For example, some participants wanted to integrate exercise into ADL, whereas others wanted more formal timeslots to complete their exercise. Although imposing a set exercise regime for all participants was avoided, there were daily activities that were encouraged for all participants to complete during the week. These included mild activation of the transverse abdominis, sit-to-stand exercises, the “bird-dog”, “back saver sit up”, the “side plank” [12] a walking programme and stretches. All these were adapted according to ability, with patients encouraged to set their own weekly goals with support. These were then reviewed at the start of the following week’s instructor-patient discussion. All home activities were progressed and adapted over duration of the programme. Participants were encouraged to utilise the back saving techniques when completing ADL and troubleshoot any personal movement difficulties that they encountered (such as lifting, getting into and out of vehicles, vacuuming, occupational tasks). At the end of week one, the group was briefed on the benefits of walking, and each participant was given a “Pedometer Challenge” recording sheet and a pedometer. For the remainder of the programme the patients were asked to record how many steps they completed each day, and new targets were mutually agreed each week.

### 2.2. Week Two—Motion Patterns and Core Activation

The main focus of this session was core activation and movement motion patterns. After a small group discussion concerning the previous week’s activity, an introduction to core activation and chair based activities were completed. These included sit-to-stands, glut activation, calf raises and abdominal bracing. Back saving motion patterns were practiced including the hip hinge and pelvic mobility applied to normal ADL such as lifting, lowering, vacuuming, pushing and pulling, sitting and general domestic tasks. The group was also differentiated into three ability groups (red, amber and green) determined through a combination of patient self-assessment and instructor observation. These sub groups were invited to partake in an outside walk focusing on posture and technique. The patients were then introduced to Nordic Walking, again focusing on mastery of technique, co-ordination and posture. Core strengthening activities were completed at the end of the session. Weekly targets were then personalized for each patient, who were advised to explore places in their local area that could be used for physical activity. Patients were also encouraged to consider significant others to share in these physical activities (spouse, children, grandchildren and friends).

### 2.3. Week Three—Aerobic Fitness

Session three focused on aerobic exercises and lifestyle management. Following a recap on posture and core activation, relaxation techniques were introduced. Patients were then inducted into the aerobic ergometers in the fitness suit (treadmill, cycle, cross-trainer, rower). Following an extended warm-up, a variety of intensities were explored to enable patients to experience light and moderate intensity exertion. Patients were encouraged to notice their breathing patterns and heart rate as well as ratings of perceived exertion. Exercise bouts were limited to 10 min on each ergometer with an emphasis on mastery, posture, technique and peer-to-peer interaction and enjoyment. The session concluded with core strengthening and a series of lower limb and lumbar stretches. In all activity blocks, patients were encouraged to select the most appropriate activity for them and where appropriate, each was adapted accordingly. Home based tasks and personalized goals were discussed alongside an emphasis on patient achievements and progress.

### 2.4. Week Four—Muscular Strength and Endurance

Following improvements in group dynamic patients were more inclined to share personal experiences. This session included discussions around diet and nutrition and patients were instructed to keep a week-long food diary for analysis the following week. The group was then introduced to a range of exercises for upper, lower and core exercise that could be completed at home with commercially available resistance bands. The aim was to ensure core stability when completing a series of balanced multi-joint, functional exercises simulating lifting, lowering, pushing and pulling. The group were then tasked with completed their own aerobic warm-up on their desired ergometer in the fitness suit, followed by an induction onto machine based multi-joint resistance equipment. Once again the emphasis was on technique, a selection of balanced exercises and correct breathing. A muscular endurance exercise prescription was adopted. Patients were then encouraged to lead their own core and flexibility activity, based on their prior learning during the programme. A review of home based activities and personalised goal setting concluded the session.

### 2.5. Week Five—Freeflow

The main purpose of week five was for patients to have a high degree of autonomy and choice. Patients could self-select activities they had already experienced on the programme, participate in an aqua based session, experience exergaming or gentle sporting options such as table tennis. The group shared feedback and comments on their food diaries. The “eat well” plate and other nutritional guidance was discussed with particular emphasis on hydration, dieting and processed food types high in fat, sugar and salt. Further group discussions also focussed on exit progamme opportunities and activities relevant to patient’s local area and preferences.

### 2.6. Week Six—Exit Programme and Post Testing

Post-testing was completed in the final session replicating week one. This enabled patients an opportunity to discuss their progress and exit strategy. The session ended with an informal discussion between the patients and instructors in a café. The patients were encouraged to continue with what they had learned, recognize the progress they had made and adhere to a physically active lifestyle.

### 2.7. Treatment of Data

All data for pre and post test results were represented as means ± standard deviation. Data were inputted and stored in a Microsoft Office Excel 2007 Spreadsheet (Microsoft Corporation, Reading, UK). Statistical analysis was performed using SPSS Software (SPSSv15 Inc., New York, NY, USA). Differences between means ± standard deviation (SD) were identified using paired sample *t*-tests where significance was accepted at *p* < 0.05. Pearson’s Correlation Coefficient were conducted to represent relationships between the change in physical measures and the MODQ.

## 3. Results

Analysis of the MODQ as identified in Table 2, revealed improvements in seven of the ten measured categories, with the greatest in sleeping (−50%), employment/homemaking (−27%) and sitting (−27%) with the overall disability rating decreasing by 16%. However none of the categories had statistically improved compared to pre-programme values. There were no improvements in the personal care and walking categories with standing increasing by 11%.

The WB12-Q, as reported in Table 3, revealed significant (*p* < 0.05) improvements for negative wellbeing −32% and although not significant (*p* > 0.05), increases in “energy” (35%) and “general wellbeing” (20%) were also identified.

**Figure 1:**
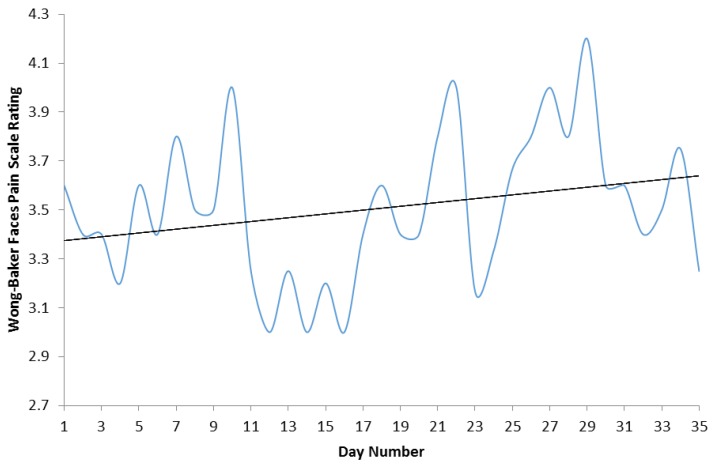
Mean Adapted Faces Pain Scale ratings from day one to day thirty five.

The value of the trendline identified in Figure 1, demonstrated a small improvement from 3.37 on day one compared to 3.60 on day thirty five. This can be extrapolated to a 7% decrease of pain reported by patients over the duration of the programme.

All measures of physical fitness improved during the six-week programme with significant (*p* < 0.05) findings in back (23%), hand grip strength (15%) and aerobic fitness (23%). Improvements in static muscular endurance (33%) and leg strength (29%) were also notable, as identified in Table 4.

The results from the body composition analysis, illustrated in Table 5, revealed significant (*p* < 0.05) 7% increase in lean mass and 13% decrease in body fat mass (*p* < 0.05) whilst body fat percentage also decreased by 9%.

**Figure 2:**
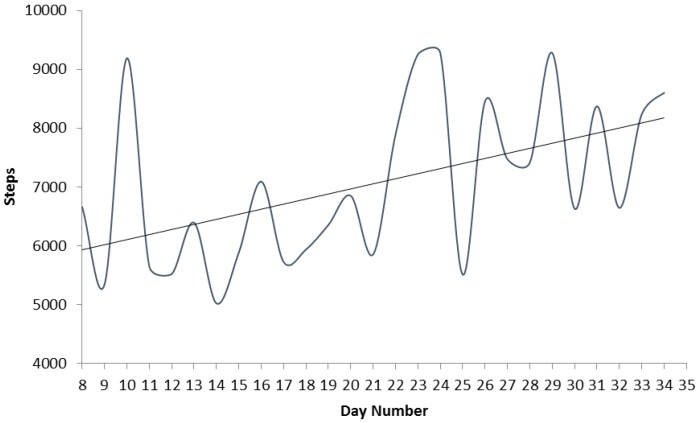
Daily mean step count of the group.

Physical activity levels as measured by daily Pedometer step count increased during the programme (Figure 2). The lowest mean number of steps completed was 5021 on day fourteen, with the greatest mean number of steps, 9281, completed on day twenty nine.

Table 6 shows a significant negative correlation between aerobic fitness and the MODQ. No other variables were related to changes in MODQ.

## 4. Discussion

The results from the MODQ indicate that although the disability of the group decreased by 16%, patients disability rating remained classified as moderate. General wellbeing was shown to increase by 20%, however neither of the net improvements in disability or wellbeing were significant (*p* > 0.05). Each patient improved their aerobic fitness, resulting in a significant (*p* < 0.05) group improvement. Data from the pedometers indicated that the group gradually increased their daily mean step count. Improvements occurred in all of the physiological performance measures, with significant (*p* < 0.05) improvements occurring in back and hand grip strength. Daily assessment of pain, using an adapted faces pain scale, highlighted that the group generally experienced less pain (higher score), as the programme progressed. Positive changes were also observed in the body composition of the group with significant (*p* < 0.05) decreases in body fat and increases in lean mass identified.

The results from the MODQ (Table 2) suggest that the intervention programme was effective at decreasing the disability of the group. Prior to the intervention programme, the mean group disability rating of 34 points placed the group closer to the “severely disabled” category (41 points). The 16% decrease in disability over the course of the programme resulted in a post-intervention disability rating of 28.4 points, which placed the group closer to the “minimally disabled” category score (20 points). These findings are encouraging given that the MODQ has been shown to be a reliable LBP questionnaire when detecting changes in disability [24]. However, it should be noted that the overall classification of ‘moderately disabled’ remained unchanged, and a decrease of less than six points (34−28.4) is at best modest. Perhaps this finding reflects the need to increase the programme’s duration to extend beyond six-weeks, the small sample of the pilot and the variable nature of non-specific LBP. An objective of the programme was to improve patient’s ability to self-manage back pain and promote exercise as an alternative to prescribed pain medication. Any large reductions in pain are likely to occur over a longer time period, emphasizing the need to conduct a patient follow up.

The overall disability classification includes pain scores from the ten categories within the MODQ. Two of the three areas which showed the greatest improvement were employment/homemaking (−27%) and sitting (−27%). These considerable improvements could be attributed to the educational approach of the programme that was designed to relate to ADL. The programme specifically addressed how to employ good posture and motion patterns such as sitting, vacuuming, opening doors and bending [12].

The only category to reflect an increase in pain was the standing category, which increased by 11%. Throughout the programme, considerable effort was dedicated to increasing overall physical activity, balance work and walking and as such this additional loading may partly explain the 11% increase in “standing pain”.

The groups’ aerobic fitness improved significantly (*p* < 0.05) over the duration of the intervention from 30.2 mL O_2_·kg^−1^·min^−1^ at pre-testing to 37.0 mL O_2_·kg^−1^·min^−1^ at post-testing. Evidence from other studies [17,18,19] indicates aerobic exercise as one of the most beneficial forms of exercise for people with LBP. Although our pilot intervention was 50% shorter than others [19], the results demonstrated promise. Our pilot recorded a 16% decrease in disability (compared to 31%), a 25% decrease in pain intensity (compared to 41%) and a significant (*p* < 0.05) 32% decrease in negative wellbeing (compared to 35% decrease in psychological strain). A large proportion of this intervention focused on promoting aerobic fitness and physical activity mediated through person-centered lifestyle changes. The “pedometer challenge”, prescription of aerobic exercise, educational sessions and self-mediated goal setting are likely to be the reasons for the significant (*p* < 0.05) group improvement in both aerobic fitness and physical activity.

Improvements in each participant’s aerobic capacity were negatively correlated (*p* < 0.05) with changes in their MODQ score. Although the sample size in this pilot demand cautious interpretation, the suggestion that greater improvements in aerobic fitness may lead to greater decreases in LBP is consistent with other more substantial studies [25]. The decrease in pain experienced by patients in our pilot was also reflected by 7% improvement identified by the adapted faces pain scale, as highlighted in Figure 1. Upon close scrutiny the lowest scores on the faces pain scale (highest pain) appear to coincide with the lowest pedometer step count (days 11–15 on Figure 1 and Figure 2). Although potentially coincidental, this supports the relationship identified between increased step count and reductions in MODQ disability.

There are various reasons why LBP patients may experience reduced pain following increased aerobic exercise. It is well established that endurance exercise can increase lipid metabolism and if the correct energy balance is established through dietary intake, a reduction in body fat percentage can occur. Our body composition data support this with percentage body fat decreasing by 9%. Excess body fat can add unnecessary loading to the spine, exacerbating pain, which can also further reduce physical activity and thereby create a cycle of deterioration. Therapies that can reduce spinal loading and promote muscle conditioning, in this case as a consequence of increased physical activity, should feature as part of a LBP exercise programme. Moreover it is well documented that aerobic exercise can increase the release of endorphins which can inhibit pain through stimulation of the central and peripheral opiate receptors [26]. Our efforts to promote aerobic physical activities that relate to ADL and functional movement, such as walking, appear justified, particularly as augmented walking programmes have been associated with reduced pain and improvements psychological health [17].

Table 3 shows that the group expressed a significant (*p* < 0.05) 32% decrease in negative wellbeing, which is comparable to the 35% decrease in psychological strain exhibited in other research [19]. Negative wellbeing is a reflection of external factors such as health and social support. The lower the score for negative wellbeing the better the patients feel about their health and the social support available. The significant decrease in negative wellbeing could reflect the effectiveness of the pilot on improving patients’ quality of life. Our person-centered approach, willingness of instructors to listen to patient experiences, and patient peer support, were intended to create a comfortable and supportive environment. Nevertheless the lack of improvement in positive wellbeing may also reflect the need for a longer intervention, as the all-encompassing nature of non-specific LBP is notoriously difficult to resolve in a short time.

The greatest improvement represented by the WB12-Q came in the “energy” category, which improved by 35%. This would indicate the group was feeling fresher, more rested and active at the end of the intervention. This finding is supported by the MODQ results for the “sleeping” category which suggest the patients had experienced a 50% improvement in their ability to sleep. These three variables were combined to produce a net 20% increase in “general wellbeing”. The “daily diary” provided the patients with the opportunity to comment about each day and the associated impact of LBP. At the start of each session the instructors would review the diaries and discuss key points with the patients. This provided a valuable means of understanding each individual patient need. For example, when planning the skill based activities the instructors used the daily diaries to match pertinent activities for individual patients (such as golf, bowls and netball). Some required personalized support for completing housework activities, yet others required advice for office and computer work. Thereby, the daily diaries enabled instructors to address patient’s specific needs and served to monitor and advise on physical activity that they were completing at home. Unpopular or ineffective exercises could also be removed or modified. The diversity of the group with regards to age and severity of LBP was managed through sub-grouping of the patients into ability levels using a traffic light system [27].

Despite the comparatively short six-week time frame, all of the measures of strength, muscular endurance and flexibility improved during the pilot. The greatest improvements were observed in static muscular endurance (33%) and leg strength (29%). Significant improvements (*p* < 0.05) occurred in aerobic fitness, back strength (23%) and hand grip strength of the left hand (15%). The improvements in the fluid goniometer test (19%) and sit and reach test (10%) were not related to MODQ scores which is consistent with other studies [16]. Nevertheless improvements in flexibility to increase the range of motion around the pelvis, hamstrings and low back would intuitively provide improvements in functionality.

The welcome improvements in grip, leg and back strength complemented the back saving techniques taught throughout the programme. The combination of educational and physical training are likely to translate to better patient ability to spare the back during lifts, loads and carrying out ADL more safely. Additionally, the improvements in muscular endurance could delay the onset of fatigue within the muscles associated with the patients’ LBP, thereby delaying the symptoms of LBP that hinder functionality during the day.

Patients were complementary about their experience during the programme and although not unexpected, patients reported that the programme was enjoyable and effective. These patient views were perhaps substantiated by the 100% retention and attendance rate recorded during the pilot.

## 5. Conclusions

The findings of this pilot suggest that our person-centered, multidisciplinary approach to group exercise may be effective at treating LBP, although six weeks may be too short to invoke substantial decreases in disability as measured using the MODQ. Future longitudinal studies, with larger samples are required for these findings to be substantiated. Improvements in aerobic fitness and physical activity may relate to the small decreases in pain (MODQ, adapted faces pain scale), disability (MODQ) and negative wellbeing (WB12-Q).

## Figures and Tables

**Table 1:** Summary of programme content.

	Theme	Activity 1	Activity 2	Activity 3	Activity 4	Activity 5	Activity 6	Activity 7
**Week One**	Introduction & Baseline	Introduction to the programme; Administration	Core activation & posture; chair based warm-up/mobility	Chester step test or alternative & education	Body composition assessment & education	Core flexion extension endurance & education	Flexibility and cool down & education	Pedometer challenge, Personalised goal setting
**Week Two**	Motion patterns and core activation	Small group discussion of daily diary, pedometers.	Chair based warm-up; sit to stands; calf raises; balance work; glut activation	Back saving motion patterns; hip hinge in context of daily tasks; explore neutral spine	Outside walk focusing on technique, pace, core activation and posture	Introduction to Nordic Walking focusing on co-ordination	Core strengthening; introduction to bird-dog, back saver sit up and side-plank	Flexibility of major muscle groups; Personalised goal setting
**Week Three**	Aerobic Fitness	Small group discussion of daily diary, pedometers. Larger group sharing as appropriate	Relaxation techniques: Lifestyle integration of learnt skills	Induction to fitness gym and aerobic equipment & education	Explore aerobic equipment; 5–8 min on up to 4 different ergometers	Progressions of bird-dog, back saver sit up and side-plank; glut max and med strengthening	Flexibility of major muscle groups;	Personalised goal setting. Review of achievements since starting the programme
**Week Four**	Muscular Strength and Endurance	Small group discussion of daily diary, pedometers. Larger group sharing as appropriate. Larger group sharing as appropriate	Introduction to resistance bands for home use	Nutrition and healthy food discussion. Food diary task	Aerobic warm up—patient led based on learnt exercise principles & increased self-efficacy	Introduction to resistance equipment in the fitness gym & education	Patient led core and flexibility exercises. Trouble shooting and adaptations	Personalised goal setting. Reflect on individualised physical activity and lifestyle management
**Week Five**	Free flow: Water, land & Exergaming	Small group discussion of daily diary, pedometers. Larger group sharing as appropriate	Analysis of food diaries and group comments/observations	Aqua aerobics or land based options: Exercise gaming; aerobic exercise; Pilates; Nordic walking; Resistance exercise; fitness suite; flexibility; Floor based exercises (bird-dog, back saver sit up and side-plank; glut max and med strengthening)			Discussion around exit programme options. Barriers to exercise	Personalised goal setting.
**Week Six**	Summary & retest	Small group discussion of daily diary, pedometers. Larger group sharing as appropriate	Retest baselines measures Chester step test; Body composition assessment; Core flexion extension; Questionnaires;			Café Group discussion Programme reflections Future plans and back pain management		Finish

**Table 2:** Pre-post programme Modified Oswestry Low Back Pain Disability (MODQ) scores (±SD) and percentage changes.

Category	Pre-Programme (±SD)	Post-Programme (±SD)	Change (%)
Pain Intensity	1.6 (1.5)	1.2 (1.6)	−25
Lifting	2.2 (1.8)	1.8 (2.1)	−18
Sitting	2.2 (0.8)	1.6 (0.9)	−27
Personal Care	0.6 (0.9)	0.6 (0.6)	0
Walking	1.2 (1.6)	1.2 (1.6)	0
Standing	1.8 (1.3)	2.0 (1.4)	+11
Sleeping	1.2 (1.3)	0.6 (0.9)	−50
Travelling	2.0 (1.0)	1.8 (0.8)	−10
Social Life	2.0 (1.2)	1.8 (1.1)	−10
Employment/Homemaking	2.2 (0.8)	1.6 (0.9)	−27
Disability Rating	34.0 (22.5)	28.4 (17.6)	−16

**Table 3:** Pre-post programme Well-being (WB12-Q) scores (±SD) and percentage changes.

Category	Pre-Programme (±SD)	Post-Programme (±SD)	Change (%)
Negative Wellbeing	5.0 (5.0)	3.4 (3.8)	−32 *
Energy	5.2 (4.2)	7.0 (2.5)	+35
Positive Wellbeing	5.4 (2.7)	5.6 (2.9)	+4
General Wellbeing	17.6 (10.2)	21.2 (8.1)	+20

* Indicates significantly different to pre-programme values (*p* < 0.05).

**Table 4:** Pre-post programme physiological performance data.

Measure	Pre-Programme (±SD)	Post-Programme (±SD)	Change (%)
Back Strength (kg)	59.0 (51.2)	72.7 (55.0)	+23 *
Leg Strength (kg)	91.6 (45.7)	118.40 (58.4)	+29
Hand Grip Strength—Left (kg)	30.0 (11.6)	34.5 (12.8)	+15 *
Hand Grip Strength—Right (kg)	32.0 (11.9)	34.6 (9.9)	+8
Prone Leg Raise (s)	56.2 (43.0)	49.8 (39.2)	+6
Plank (s)	35.3 (25.5)	53.75 (45.5)	+33
Fluid Goniometer (°)	52.7 (17.1)	62.67 (11.2)	+19
Sit and Reach (cm)	21.7 (9.7)	23.92 (9.0)	+10
Aerobic Capacity (mL O_2_·kg^−1^·min^−1^)	30.2 (7.60)	37.0 (4.5)	+23 *

* Indicates significantly different to pre-programme values (*p* < 0.05).

**Table 5:** Pre and post programme anthropometric measures.

Measure	Pre-Programme (±SD)	Post-Programme (±SD)	Change (%)
Body Fat Percentage (%)	32.5 (7.8)	29.6 (10.1)	−9
Body Fat Mass (kg)	27.4 (6.4)	23.8 (7.31)	−13 *
Lean Mass (kg)	54.5 (16.0)	58.1 (17.5)	+7 *
Total Mass (kg)	87.7 (23.1)	88.6 (22.9)	+1

* Indicates significantly different to pre-programme values (*p* < 0.05).

**Table 6:** Relationship between the change in MODQ and physiological data.

Mean Change in Physical Measure	*r*-Value
Body mass (Kg)	−0.141
% Body Fat	−0.615
Back Strength (Kg)	0.171
Leg Strength (Kg)	−0.365
Hand Grip (right) (Kg)	−0.107
Hand Grip (left) (Kg)	0.409
Prone Leg Raise (s)	−0.018
Fluid Goniometer (°)	−0.176
Sit and Reach (cm)	−0.614
Aerobic Capacity (ml sO_2_/Kg/min)	−0.973 *
Walking (steps per day)	−0.179

* Indicates correlation is significant *p* < 0.05 (2-tailed).

## Abbreviations

The following abbreviations are used in this manuscript:

MODQ	Modified Oswestry Disability Questionnaire
LBP	Low back pain
WB12-Q	Well-Being 12 Questionnaire
ADL	Activities of Daily Living
